# Longitudinal Speech Changes in Velopharyngeal Function in Submucous Cleft Palate

**DOI:** 10.1002/ohn.1218

**Published:** 2025-03-10

**Authors:** Soukaina Eljamri, Randall Harley, Matthew Ford, Noel Jabbour

**Affiliations:** ^1^ University of Pittsburgh School of Medicine Pittsburgh Pennsylvania USA; ^2^ Department of Otolaryngology University of Pennsylvania Philadelphia Pennsylvania USA; ^3^ UPMC Children's Hospital of Pittsburgh Pittsburgh Pennsylvania USA

**Keywords:** furlow palatoplasty, Pittsburgh Weighted Speech Score, speech, submucous cleft palate, velopharyngeal dysfunction

## Abstract

**Objective:**

To evaluate longitudinal speech changes in surgical and nonsurgical patients with submucous cleft palate (SMCP) and velopharyngeal dysfunction (VPD).

**Study Design:**

Retrospective cohort study.

**Setting:**

Single academic medical center.

**Methods:**

In total, 204 patients with documented SMCP and VPD from January 2002 to 2008 were identified. Using a multilevel mixed‐effects model, we examined the association between surgical status and speech score trajectories, adjusting for age, sex, race, and severity.

**Results:**

In total, 204 children were included (mean [SD] age, 4.9 [3.1] years; 124 [60.8%] male; 114 [55.9%] furlow palatoplasty). Amongst surgical patients, the postsurgical median baseline score was 4 and scores were predicted to continue to decrease over time at a rate of 0.04 points per year (coefficient [*β*] −0.04, 95% confidence interval [95% CI] −0.20 to 0.13, *P* = .64). Presurgical speech scores were predicted to decrease over time at a rate of 0.78 points per year (*β* −0.78, 95% CI −1.14 to −0.41, *P* < .001). With a median presurgical score of 14, it would take 9 years to achieve normal speech scores without surgical intervention. In nonsurgical patients, speech scores were predicted to decrease at a rate of 0.23 points per year (*β* −0.23, 95% CI −0.51 to 0.04, *P* = .094).

**Conclusion:**

Speech production in VPD can improve over time without surgical intervention but is not expected to do so within the critical window for speech development. Surgical intervention improves speech by rates of change that cannot be achieved without surgery.

Submucous cleft palate (SMCP) involves impaired median fusion of the muscles of the soft palate and is characterized by Calnan's triad: zona pellucida, bifid uvula, and notching of the hard palate.^1^ There is a spectrum of severity in SMCP requiring subclassification as overt or occult. An overt SMCP has one or more of the triad features, whereas occult has similar palatal muscle diastasis in the absence of the classic triad features.^2^ Approximately half of all SMCP patients are asymptomatic, whereas the other half may present with speech deficits and/or middle ear disease.^2^, ^3^

Velopharyngeal dysfunction (VPD) is a consequence of SMCP that is defined by inadequate closure of the soft palate and pharynx to separate the oral and nasal cavities, resulting in hypernasal speech. Surgical intervention for correction of SMCP is usually indicated for correction of these speech deficits, which may be evaluated by various metrics, including nasoendoscopy, multiview video fluoroscopy, magnetic resonance imaging, and nasometry. The Pittsburgh Weighted Speech Score (PWSS), first described in 1979, has been used as a validated measure to evaluate VPD on a quantitative scale, using the following categories: nasal air emission, presence of facial grimace, nasality/resonance, phonation/voice, and articulation.^4^ Subjective perceptions of VPD may also have an impact on communication, socialization, and behavioral development and may also impact management decisions. In a 2013 study, researchers evaluated the perception of hypernasal speech and social judgments of individuals with hypernasal speech by their school‐aged peers. They found that patients with even mild hypernasality were perceived by peers to be less likely to “fit in with friends” or “get good grades” and more likely “to be teased.”^5^

There is currently no consensus on the recommended timing of surgical intervention for patients with SMCP and VPD as there is tremendous variability in clinical presentation and severity.^3^, ^6^, ^7^, ^8^ Often, the nuance of this decision is left to the clinical judgment of the care team and preferences of the family. However, given the time‐sensitive nature of speech development, it is important to consider the consequences of delayed intervention and potential compensatory mechanisms that may develop in the event of persistent VPD. There are currently no studies that evaluate the longitudinal trajectory of VPD in children with SMCP. This information could be useful when deciding whether it is reasonable to delay or forgo surgical intervention. In this study, we analyzed baseline VPD severity using PWSS and evaluated longitudinal changes in speech scores among both surgical and nonsurgical patients with diagnosed SMCP.

## Materials and Methods

This is a retrospective analysis from a single tertiary academic center of patients born between the years 2002 and 2013. Patients were recruited from this time range to allow for adequate longitudinal follow‐up. Among surgical patients, the year of surgery ranged from 2004 to 2018. All patients included had a diagnosis of SMCP and VPD, and a follow‐up period of at least 2 years. For nonsurgical patients, follow‐up period was measured starting from time of diagnosis, and for surgical patients, follow‐up period was measured starting from time of surgery. The primary goal was to compare longitudinal changes in speech production between surgical and nonsurgical patients. Speech production was measured at each follow‐up appointment using the PWSS. The scoring guidelines are as follows: scores of 1 to 2 indicate borderline competence, scores of 3 to 6 indicate borderline incompetence, and scores greater than 7 indicate velopharyngeal incompetence. For this analysis, we defined a speech score of 7 or greater as indicative of stigmatized speech with nasal air emission and hypernasality, meaning speech deficits can be appreciated by peers and untrained lay persons. We defined scores below 3 as normal speech. Additional variables measured in this study included age, gender (male, female), race (white, black, Asian, and Indian), SMCP severity (occult, overt), and diagnosis of a genetic syndrome (yes, no). Genetic syndromes included 22q deletion, plagiocephaly, hemifacial microsomia, and craniosynostosis. Overt SMCP severity was defined by documented evidence of a zona pellucida, bifid uvula, and notching of the hard palate. Although occult SMCP severity was assigned to patients who may have exhibited some, but not have all three, of these features.

All statistical analyses were performed using STATA SE 17.0 for Mac OS. Descriptive statistics, including proportions, means, and standard deviations (SD), were used to compare demographic and clinical features between treatment groups. The primary goal was to compare changes in speech production over time between surgical and nonsurgical patients. Having repeat PWSS measurements for each patient gives rise to the issue of nonindependence, as measurements for an individual patient will be more similar than measurements between patients. Using a traditional linear regression model (mixed‐effects model), independence is achieved by aggregating all values for an individual patient, which prevents evaluation of longitudinal progression. This can be overcome using a multilevel mixed‐effects model, in which separate regression lines are created for individuals and then averaged across patients and treatment groups. The model can be expressed:

$$y_{\mathrm{ij}}=\beta_{1}+\beta_{2}x_{\mathrm{ij}}+\zeta_{1j}+\zeta_{2j}x_{\mathrm{ij}}+\varepsilon_{\mathrm{ij}}$$

where *y*_*ij*_ is the value of the response variable (PWSS) for the *j*th of $n_{i}$ observations (patient) in the *i*th of M groups or clusters (treatment group). $\beta_{1}$ is the overall mean intercept and $\beta_{2}$ is the overall mean coefficient. $x_{\mathrm{ij}}$ is the fixed‐effect regressors (patient age) for observation *j* in group *i*. $\zeta_{1j}$ is the intercept deviation from the mean and $\zeta_{2j}$ is the slope deviation from the mean for observation *j*. $\varepsilon_{\mathrm{ij}}$ is the error for observation *j* in group *i*. For this model, there were fixed‐effects with respect to the predictors current age, age at diagnosis, gender, race, presence of syndrome, and SMCP severity. There were random effects with respect to individual and treatment group. This study was reviewed and approved by the University of Pittsburgh Institutional Review Board (STUDY19100247). Study data were collected and stored using the Research Electronic Data Capture (REDCap) software hosted at a single institution.

## Results

The final analysis included 204 pediatric patients diagnosed with SMCP and VPD. Nearly half of the patients were in the nonsurgical group (44%; n = 90), whereas the remainder were in the surgical group (56%; n = 114). Patients in the nonsurgical group tended to be older at the age of diagnosis compared to patients in the surgical group (5.7 vs 4.2 years; *P* < .001). Both groups were predominantly male and white. Children in the nonsurgical group were more likely to have occult SMCP (67.8% vs 50%; *P* = .015), whereas those in the surgical group were more likely to have SMCP in the setting of a genetic syndrome (66.7% vs 52.2%; *P* = .044). At the time of diagnosis, the surgical group had a presurgical median PWSS of 14 (interquartile range [IQR] 12), whereas the nonsurgical group had an initial PWSS of 4 (IQR 4). The mean length of follow‐up was 3.94 years (SD 3.15) in nonsurgical patients and 6.79 years (SD 3.53) in surgical patients (*P* < .001). Demographics and clinical characteristics are summarized in Table 1.

**Table 1:** Demographics and Clinical Characteristics

	Nonsurgical	Surgical	
Variables	n = 90	n = 114	*P*‐value^a^
Age at diagnosis, y, mean (SD)	5.7 (3.6)	4.2 (2.4)	**<.001**
Gender, n (%)			.25
Female	31 (34.4)	49 (43.0)	
Male	59 (65.6)	65 (57.0)	
Race, n (%)			.94
White	86 (95.6)	106 (93.0)	
Black	3 (3.3)	5 (4.4)	
Asian	1 (1.1)	2 (1.7)	
Indian	0 (0)	1 (0.9)	
SMCP severity, n (%)			**.015**
Occult	61 (67.8)	57 (50.0)	
Overt	29 (32.2)	57 (50.0)	
Genetic syndrome, n (%)			**.044**
No	43 (47.8)	38 (33.3)	
Yes	47 (52.2)	76 (66.7)	
Initial PWSS, median (IQR)	4 (4)	14 (12)	**<.001**

Abbreviations: IQR, interquartile range; n, sample size; PWSS, Pittsburgh Weighted Speech Score; SD, standard deviation; SMCP, submucous cleft palate; y, years.

^a^ Values with *P*‐value <.05 labeled in bold.

**Figure 1:**
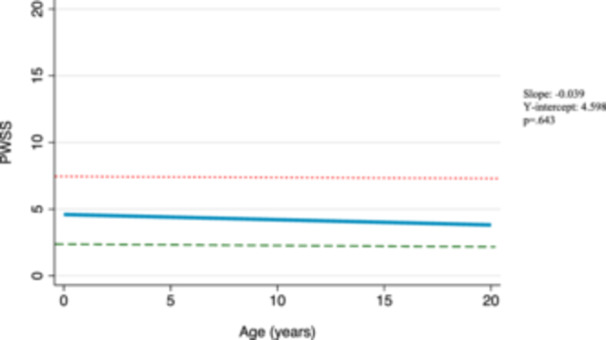
Predicted Pittsburgh Weighted Speech Score (PWSS) changes in surgical patients after surgery. Mixed‐effects model graphical representation of the predicted PWSS trajectory among surgical patients after surgery. A score of 7 is indicated by a dotted red line and a score of 3 is indicated by a dashed green line. The slope ($\beta_{1}$), *y*‐intercept ($\beta_{0}$), and *P*‐value from mixed‐effects model are displayed on right‐hand side of the figure.

**Figure 2:**
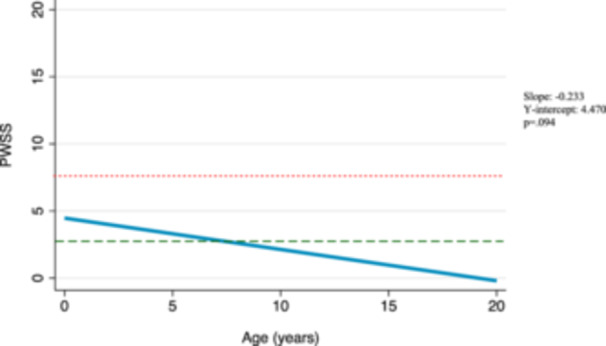
Predicted Pittsburgh Weighted Speech Score (PWSS) changes in nonsurgical patients. Mixed‐effects model graphical representation of the predicted PWSS trajectory among nonsurgical patients. A score of 7 is indicated by a dotted red line and a score of 3 is indicated by a dashed green line. The slope ($\beta_{1}$), *y*‐intercept ($\beta_{0}$), and *P*‐value from mixed‐effects model are displayed on right‐hand side of the figure.

**Figure 3:**
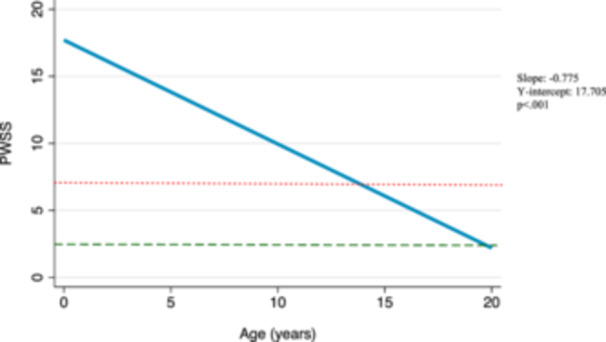
Predicted Pittsburgh Weighted Speech Score (PWSS) changes in surgical patients before surgery. Mixed‐effects model graphical representation of the predicted PWSS trajectory among surgical patients before surgery. A score of 7 is indicated by a dotted red line and a score of 3 is indicated by a dashed green line. The slope ($\beta_{1}$), *y*‐intercept ($\beta_{0}$), and *P*‐value from mixed‐effects model are displayed on right‐hand side of the figure.

Amongst surgical patients, the postsurgical median baseline score was 4, compared to a presurgical median baseline of 14. According to our model, these scores are predicted to continue to decrease over time at a rate of 0.04 points per year (*β* −0.04, 95% confidence interval [95% CI] −0.20 to 0.13, *P* = .64) (Figure 1). This trend, however, is not statistically significant. We also considered longitudinal changes in speech scores in the cohort of nonsurgical patients and found that without surgical intervention, speech scores in this group are also predicted to decrease over time at a rate of 0.23 points per year (*β* −0.23, 95% CI −0.51 to 0.04, *P* = .094) (Figure 2). This trend is also not statistically significant. Further, we considered predicted changes in surgical patients had they not received surgery using trends of their presurgical scores over time. These presurgical speech scores were predicted to decrease over time at a rate of 0.78 points per year (coefficient [*β*] −0.78, 95% CI −1.14 to −0.41, *P* < .001) (Figure 3). In evaluating this trend with respect to the median baseline presurgical speech score of 14, our model predicts that it would take 9 years for patients to achieve normal speech scores without surgical intervention. Graphical representations of these trends are shown in 1, 2, 3.

**Figure 4:**
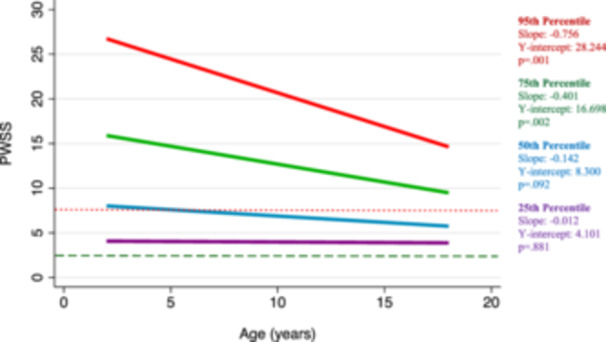
Predicted Pittsburgh Weighted Speech Score (PWSS) changes by baseline percentile. Mixed‐effects model graphical representation of the predicted PWSS trajectories, with respect to baseline score percentiles, among nonsurgical and presurgical patients. The 25th percentile (PWSS = 4) is shown in purple, the 50th percentile (PWSS = 8) is shown in blue, the 75th percentile (PWS = 16) is shown in green, and the 95th percentile (PWSS = 27) is shown in red. A score of 7 is indicated by a dotted red line and a score of 3 is indicated by a dashed green line. The slope ($\beta_{1}$), *y*‐intercept ($\beta_{0}$), and *P*‐value from mixed‐effects model are displayed on right‐hand side of the figure.

In our longitudinal analysis stratified by initial PWSS percentile, we found that patients with higher initial PWSS have faster improvement in speech. Patients within the 95th percentile of initial speech scores (PWSS = 27) are estimated to improve at a rate of 0.76 points per year (*β* −0.76, 95% CI −1.21 to −0.31, *P* = .001), whereas patients within the 75th percentile of initial speech scores (PWSS = 16) are estimated to improve at a rate of 0.40 points per year (*β* −0.40, 95% CI −0.66 to −0.14, *P* = .002). These trends are graphically represented in Figure 4.

## Discussion

In this study, we found that patients with higher baseline speech scores, overt SMCP, and underlying genetic syndromes were more likely to undergo surgery for correction of SMCP. Nonsurgical patients had lower baseline speech scores and predominantly occult SMCP. Surgical patients had a significantly longer length of follow‐up than nonsurgical patients, likely because postoperative care requires a longer period of follow‐up for assessment by otolaryngology and speech language providers. Through evaluation of PWSS over a greater than 2‐year period, we built a longitudinal model with up to 20 years of predicted change over time. Although PWSS is predicted to gradually improve over time for postsurgical and nonsurgical patients, only presurgical patients demonstrate a statistically significant trend toward normal speech. Unfortunately, our model also indicated that without surgical intervention, these patients are unlikely to achieve normal speech within the critical window for speech development. When considering predicted speech changes with respect to baseline PWSS percentile, we found that patients with higher baseline scores are predicted to improve at a faster rate than patients with lower baseline speech scores. This analysis may be particularly useful for patients with borderline speech scores, where the timing and utility of surgical intervention may be less obvious. Overall, our study provides further evidence that management and timing of surgical intervention should consider not only baseline speech scores but also the predicted evolution of scores over time.

The critical window for speech development plays an important role when considering the timing of surgical intervention for VPD and is generally defined as the age of 18 to 24 months, as determined by the period in which children begin forming two‐word sentences.^7^ However, evaluation for speech deficits in an otherwise asymptomatic patient may not occur until much later.^6^ In a recent trial conducted across centers in Europe and South America, infants with isolated, nonsyndromic cleft palate were assigned to undergo primary corrective surgery at either 6 months or 12 months of age to determine whether timing of cleft palate correction had an impact on velopharyngeal insufficiency development at age 5. They concluded that patients with earlier correction at 6 months of age were less likely to develop velopharyngeal insufficiency at age 5.^8^ Similarly, a 2014 study of nonsyndromic patients with both submucous clefts and overt clefts found that surgical repair with furlow palatoplasty after 18 months is associated with significantly increased incidence of articulation errors.^9^ A 2017 retrospective study of nonsyndromic patients with SMCP found that surgical repair after age 4 was associated with persistent misarticulation compared to patients who underwent surgical repair before age 4.^6^ Alternatively, a 2018 retrospective study evaluated timing of surgical intervention by furlow palatoplasty in patients with SMCP and found no difference in speech outcomes between patients with early operative intervention (<4 years) and late operative intervention (>4 years).^7^

In patients with SMCP and VPD, management decisions must consider which patients would benefit from immediate surgical intervention to prevent the persistence of hypernasal speech and compensatory mechanisms in speech development and which patients may improve independent of surgical intervention. We demonstrated in this study that PWSS in all patients are predicted to improve over time independent of any intervention, regardless of baseline speech score. However, rates of change varied and determined whether patients would be expected to achieve scores within the range of normal speech within an appropriate window for speech development. The mechanism underlying the predicted improvement in speech scores seen in nonsurgical patients remains unclear. However, this may represent compensatory mechanisms or the impact of nonsurgical interventions, such as speech therapy. Although universal practice patterns do not currently exist for determining which patients are appropriate surgical candidates, we propose in this study, that consideration of longitudinal trajectories may be useful in evaluating these patients for surgery and in weighing the risks and benefits of surgical intervention versus nonsurgical management of hypernasality. These data may be helpful in counseling patients and families to assist in shared decision‐making when considering surgical intervention and the most appropriate timing.

Nevertheless, this study has important limitations. As discussed previously, each of our study groups had outlier patients with higher speech scores who did not undergo surgery and similarly, patients with lower baseline speech scores who received surgery. There are numerous factors that may impact whether or not a patient receives surgery and further, how their speech deficits may evolve over time, which could not be feasibly incorporated into our retrospective model. These may include but are not limited to parental/patient preference, socioeconomic variables, subjective perceptions of speech dysfunction, and presence of SMCP comorbidities including fistulas, regurgitation, or eustachian tube dysfunction. Another important limitation is the variability in data points between the study groups and the difference in their frequency and length of follow‐up. Nonsurgical patients had a shorter length of follow‐up than surgical patients and likely less frequent interaction with their provider in that period and thus fewer documented speech scores. We attempted to control for this variability by using a mixed‐effects model, rather than a traditional logistic regression for our analysis. A mixed‐effects model considers subject‐specific trends in speech scores and reports the average trend across all patients, thus limiting the bias introduced when consolidating varying data points from each individual patient. Similarly, with increased follow‐up and evaluation, surgical patients may have had more opportunities for recognition of underlying comorbidities that may have impacted the timing and indication for surgery. Further, data regarding secondary speech surgeries or revision surgeries in the surgical patients were not addressed, and longitudinal trends were only based on speech scores after the initial surgery. Finally, although our trends were reported linearly in graphical representations of average cohort trends over time, it is important to consider that individual trends may be nonlinear in reality and that in using a linear average, we are missing variation in rate of change over time.

## Conclusions

In patients with SMCP and VPD, speech production as determined by PWSS can gradually improve over time without surgical intervention but would not be expected to do so before the critical window for speech development closes. In patients with SMCP and VPD, according to our model, speech production as determined by PWSS can gradually improve over the course of many years without surgical intervention. As a consequence of this sluggish rate of improvement, patients receiving nonoperative management are unlikely to achieve normal speech within the critical window for speech development. Speech therapy interventions may be required long term without surgical intervention which is a substantial cost in time, resources, and payment. This should be considered when counseling families for those patients with significant alterations in articulation associated with VPD. Surgical intervention may decrease the longitudinal need of speech therapy as improved VP function may assist the child in achieving normalized articulation. Ultimately, surgical intervention improves speech scores by rates of change that cannot be achieved without surgery, especially within the critical window for speech development. Future research is necessary to further elucidate the longitudinal impact of surgical versus nonsurgical intervention on speech development in these patients.

## Author Contributions

**Soukaina Eljamri:** conceptualization, data curation, investigation, methodology, writing—original draft preparation, review and editing; **Randall Harley:** conceptualization, formal analysis, investigation, methodology, writing—review and editing; **Matthew Ford:** conceptualization, methodology, writing—review and editing; **Noel Jabbour:** conceptualization, methodology, project administration, supervision, writing—review and editing.

## Disclosures

### Competing interests

The authors have no conflicts of interest to declare.

### Funding source

The authors have no financial disclosures.
